# A pilot trial investigating the feasibility of a future randomised controlled trial of Individualised Placement and Support for people unemployed with chronic pain recruiting in primary care

**DOI:** 10.1017/S1463423622000342

**Published:** 2022-07-22

**Authors:** Karen Walker-Bone, Simon DS Fraser, Cathy Price, Nick Maguire, Cyrus Cooper, Ira Madan, Georgia Ntani, Cathy L Linaker

**Affiliations:** 1 MRC Versus Arthritis Centre for Musculoskeletal Health and Work, University of Southampton, Southampton, UK; 2 MRC Lifecourse Epidemiology Centre, University of Southampton, Southampton, UK; 3 Primary Care, Population Sciences and Medical Education, Faculty of Medicine, University of Southampton, Southampton, UK; 4 Solent NHS Trust, Southampton, UK; 5 Department of Psychology, University of Southampton, Southampton, UK; 6 Occupational Health Service, Guy’s and St Thomas’ NHS Foundation Trust, London, UK; 7 King’s College London Faculty of Life Sciences and Medicine, London, UK

**Keywords:** chronic pain, pilot, recruitment, quality of life, vocational rehabilitation

## Abstract

**Background:**

We investigated the feasibility of recruiting patients unemployed for more than 3 months with chronic pain using a range of methods in primary care in order to conduct a pilot trial of Individual Placement and Support (IPS) to improve quality of life outcomes for people with chronic pain.

**Methods:**

This research was informed by people with chronic pain. We assessed the feasibility of identification and recruitment of unemployed patients; the training and support needs of employment support workers to integrate with pain services; acceptability of randomisation, retention through follow-up and appropriate outcome measures for a definitive trial. Participants randomised to IPS received integrated support from an employment support worker and a pain occupational therapist to prepare for, and take up, a work placement. Those randomised to Treatment as Usual (TAU) received a bespoke workbook, delivered at an appointment with a research nurse not trained in vocational rehabilitation.

**Results:**

Using a range of approaches, recruitment through primary care was difficult and resource-intensive (1028 approached to recruit 37 eligible participants). Supplementing recruitment through pain services, another 13 people were recruited (total *n* = 50). Randomisation to both arms was acceptable: 22 were allocated to IPS and 28 to TAU. Recruited participants were generally not ‘work ready’, particularly if recruited through pain services.

**Conclusion:**

A definitive randomised controlled trial is not currently feasible for recruiting through primary care in the UK. Although a trial recruiting through pain services might be possible, participants could be unrepresentative in levels of disability and associated health complexities. Retention of participants over 12 months proved challenging, and methods for reducing attrition are required. The intervention has been manualised.

## Background

Chronic pain, defined as pain which persists or recurs beyond 3 months (Treede et al., 2019), is a major international health problem associated with mental illness, job loss, impaired function and poor quality of life (Tunks et al., 2008). According to the Global Burden of Disease studies, chronic pain is now the leading cause of disability and disease burden worldwide, and the size of the problem is increasing (Vos et al., 2017). Epidemiological studies suggest that chronic pain affects between one-third and one-half of the population of the UK (approx. 28 million adults) and that prevalence increases with age so that, as the population ages, higher rates are predicted (Fayaz et al., 2016). Chronic pain is commonly associated with musculoskeletal conditions such as arthritis, low back pain or fibromyalgia, but is also associated with cancer, headaches, neuropathic or idiopathic. Between 20%–27% of people of working age with chronic pain are unable to participate in their usual activities, including work, due to their pain (Donaldson, 2009). The healthcare costs associated with treating chronic pain are high but it has been estimated that as much as 48%–88% of the total cost burden of chronic pain arises from indirect costs associated with impaired productivity, sick leave, disability benefits and other aspects of work disability. Indeed, three out of the top ten conditions that impact productivity are painful disorders (back/neck pain, arthritis conditions and other chronic pain) (British Pain Society, 2011), and in a national audit, as many as 40% of people attending UK pain clinics was prevented from work (paid or voluntary) by pain (British Pain Society, 2012).

Employment can have important benefits for individuals in terms of social standing, finances and their health and wellbeing (Waddell and Burton, 2006; Black, 2008), and prolonged unemployment, for any reason, causes additional health problems (Moser et al., 1984). However, people with chronic pain face many barriers in finding employment or returning to work after sickness absence (Grant et al., 2019a; 2019b). Individualised Placement and Support (IPS) is an evidence-based model of vocational rehabilitation originally developed in the USA to help people with severe mental health conditions gain and keep employment (Drake and Becker, 1996; Drake 1998). Rates of unemployment amongst people with severe mental health conditions can be up to 95% but data from over 20 randomised controlled trials (RCTs) have shown that IPS is effective at improving employment with rates approaching 50% in this population (Burns et al., 2007). The model is based on a ‘place then train’ approach rather than traditional approaches (sheltered employment or vocational training). Recently, therefore, there have been calls to extend this approach to people with other long-term health conditions (Bond et al., 2019), including chronic pain, and some pilot work has been undertaken in Norway (Rodevand et al., 2017, Linnemorken et al., 2018) and the UK (Froud et al., 2020) recruiting people with chronic pain from established hospital-based pain services. However, the majority of UK chronic pain patients do not attend pain services as these are usually commissioned with strict entry criteria usually related to local capacity of primary care services. Therefore, in 2016, the National Institute for Healthcare Research Health Technology Assessment (NIHR HTA) programme called for applications to undertake a pilot study to investigate the feasibility of a future RCT of IPS for people recently unemployed through chronic pain, with the emphasis on recruitment of participants through primary care.

Specifically, our objectives were to (a) evaluate different approaches in primary care, to identify and recruit people recently unemployed with chronic pain; (b) define and develop a Treatment as Usual (TAU) care package for the control group and evaluate its acceptability; (c) explore the acceptability of procedures for consent, randomisation and retention for both intervention and control groups over 12 months of follow-up (including adherence to the study protocol, attrition and follow-up questionnaire completion); (d) integrate a pain management intervention with IPS (e) inform appropriate primary and secondary outcome measures for a definitive trial; and (f) develop and manualise IPS for people with chronic pain (to establish what is required, specific training needs for those providing IPS, and whether it can be delivered within the health care system in the UK).

## Methods

### Pilot trial design

The trial was a two-arm pilot RCT, comparing integrated IPS with TAU to evaluate the feasibility and acceptability of such a trial. Ethical approval was obtained through the University of Southampton Research Ethics Committee (ID 23 853) and research governance approval from the Health Regulatory Authority (17/SCA/0398) in October 2017, and the trial was registered (ISRCTN No: 30094062; Date 01/12/2016).

### Eligibility criteria

Participants were eligible if they had chronic pain (continuous pain for more than 3 months), had completed the diagnostic pathway for their condition and were not expected to recover within the next 12 months; were unemployed through chronic pain for at least 3 months; had not previously received IPS; were able to provide written, informed consent; and wanted to work.

### Identification and recruitment of eligible participants

The pilot trial was advertised through primary care research networks and educational events targeting GPs in order to recruit a diverse range of practices (ie, practices of different size, area deprivation, case mix, age range, geographical location) within the cities of Portsmouth and Southampton, England (both of which have significant population deprivation). Participating practices were asked to select at least two of the following methods to identify people with chronic pain: posters; searches of the computerised patient databases to identify those Read codes (Chisholm, 1990) (codes used to record long-term conditions and other aspects of medical encounters in Primary Care systems in England) most likely associated with chronic pain (Appendix 1); opportunistic recruitment during face-to-face appointments with a GP; or targeted recruitment after hand-searching patient records.

Supporting study materials for each method were provided by the research team, and to minimise additional work for primary care staff, any potentially interested participant was invited to return a reply-paid slip or telephone or Email the study coordinator. The study coordinator followed up all enquiries with a telephone appointment to provide any required additional information, screen an individual’s eligibility, obtain verbal consent for randomisation (using computer-generated algorithm (block 1:1)) to either the active IPS arm or the TAU control arm. Appointments were then arranged at the participants’ convenience (either with the study coordinator in the individual’s GP surgery if randomised to TAU or with the employment support worker (ESW) at the City Council premises if randomised to the IPS arm), by which point written, informed consent was obtained.

### TAU

There is no evidence-based alternative to IPS in UK healthcare but a number of services are provided by the Department for Work and Pensions, local government and the voluntary sector, to which individuals may self-refer or be referred by healthcare professionals. However, the nature and type of support varies by region, and it is unclear to what extent primary care staff are aware of, or direct patients to, these services. People with chronic pain frequently lack confidence, skills and self-efficacy as a result of their condition, which is often complicated by psychological comorbidities (De Heer et al., 2014). Therefore, a specially developed booklet was created in collaboration with a Patient and Public Involvement (PPI) Group (*n* = 6 members), to supplement primary care TAU and signpost participants to local employment and healthcare services (available from: https://www.journalslibrary.nihr.ac.uk/programmes/hta/1510802#/). We chose this approach both for ethical reasons and to encourage participation. As the intervention was provided in two cities with different regional services, bespoke TAU booklets were created for each city. As recommended by the PPI group, the booklets included blank pages for participants to make their own notes and to encourage them to construct a list of goals structured towards ultimately securing gainful employment. The booklets also promoted positive messages about self-efficacy and the value of employment in enhancing health/wellbeing.

Participants randomised to TAU were invited to one face-to-face appointment with the study coordinator (a qualified research nurse without any specialist training in vocational rehabilitation) at their GP practice. After answering any questions, obtaining written, informed consent and completion of the baseline questionnaire by the participant, the study coordinator spent approximately 10 min guiding the participant through the TAU booklet and encouraging them to take it home to read it at their leisure. Each appointment lasted approximately one hour. Travel costs were reimbursed.

### IPS

**Figure 1.**
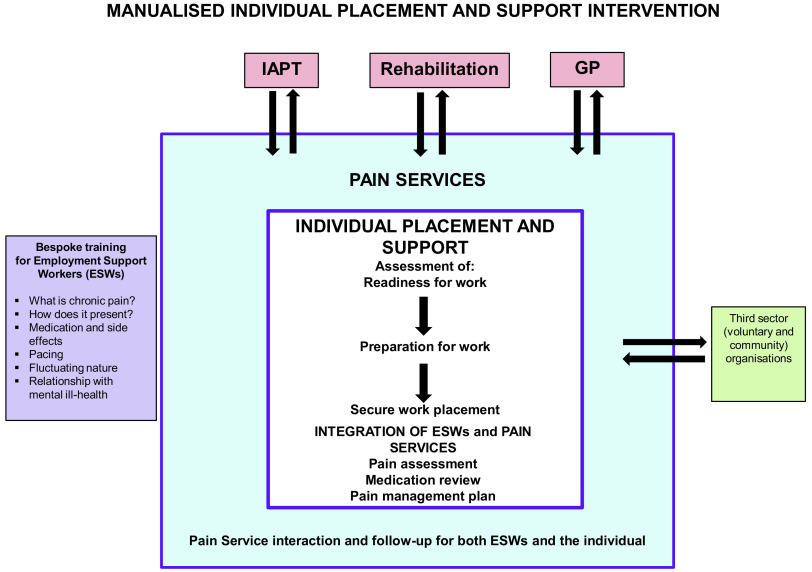
Schematic illustrating the manualised Individual Placement and Support for chronic pain patients

Two experienced ESWs already providing IPS in each city were seconded to the research team for this project. Prior to recruitment, they received training about chronic pain which included: how the condition presents in practice; the types and side effects of medication used to manage the pain; and common approaches used by pain experts to enable functioning (Figure 1).

After randomisation to IPS, each participant was telephoned by the trained ESW in their locality to invite them to attend a face-to-face appointment held at the local City Council Offices. After addressing any questions, obtaining written, informed consent to participate in the trial and IPS and asking them to complete the baseline questionnaire, the ESW started work with the individual to establish their preparedness for work. Participants in the IPS arm were also given the TAU booklet and encouraged to take it home to read it at their leisure.

All participants randomised to IPS were seen by both their assigned ESW and the community pain occupational therapist (OT) at one of their initial appointments. The focus of these joint meetings was to assess the participant’s pain and current pain management strategies, with the possibility of specific counselling and support from the OT, signposting to other relevant services or follow-up by the pain team. The ESWs were invited to integrate as much as possible with the local pain services, attend multi-disciplinary team meetings and follow-up pain service use was available to all participants in the IPS arm.

Following local procedures for IPS, and alongside people receiving IPS through a separate initiative (the Solent Jobs Programme which recruited people unemployed for more than two years with long-term health conditions), pilot trial participants met their ESW as frequently as required to support and develop their employment plans over a maximum of 12 months. Fundamentally, the process comprised three stages: assessing the individual’s preparedness for work; preparing them for work; and finally, selecting and allocating the individual to a competitive paid work placement (IPS). Travel costs incurred by participants for all visits were reimbursed.

IPS relies upon key principles, the first of which is a goal of competitive employment but the other principles are zero exclusion; attention to clients’ preferences; rapid job search; targeted job development; integration of the employment services with their healthcare; benefits counselling and individualised long-term support. There are few settings in which it is possible to set up a service that can meet these principles solely for the purposes of a pilot study. Our study benefitted from being nested in the existing Solent Jobs programme, set up 3 years previously and having engaged with > 700 local employers. Through this, pilot study participants in the IPS arm were offered competitive job placements for which they had to attend an interview (and could be not selected), but these placements offered flexibility over and above standard employment. Local employers had been engaged in providing work placements by the City councils, aiming to support them to address recruitment needs. This proved easier in some sectors including Hospitality and Warehouse and Logistics, but more difficult in others (eg, senior administration), and placements were frequently based on small- and medium-sized employers, who were attracted to the promise of local recruitment and associated their involvement in this with creating a positive image. Relevant businesses/roles were identified, and then, opportunities brokered based on participants’ circumstances and job interests. The salary of the placement worker was paid by the Council for up to 6 months with the expectation of longer-term employment beyond this timepoint.

### Data collection

Questionnaires were developed for completion at baseline and 3, 6 and 12 months of follow-up. At baseline, information was collected on demographic and lifestyle factors; qualifications; employment history; employment aspirations; comorbidities; healthcare utilisation; finances; welfare benefits; and functional health literacy. At baseline and each follow-up, we collected EQ5D-5L; Brief Pain Inventory (Mendoza et al., 2006); health care utilisation; return-to-work self-efficacy scale (Brouwer et al., 2011; Black et al., 2016; Lagerveld et al., 2016; Black et al., 2017; Black et al., 2018; Black et al., 2019); Warwick-Edinburgh Mental Wellbeing Scale (Tennant et al., 2007), PHQ-9 (Kroenke et al., 2001); self-rated health (Kondo et al., 2009), Rosenberg Self-Esteem Scale (Rosenberg, 1965) and markers of pain as recommended by the IMMPACT statement (Dworkin et al., 2005). At follow-up after 3 and 6 months, everyone was asked to report if they had done any paid work since the previous questionnaire, what type of work, over what duration (options: < 1 week; > 1 week but < 1 month, > 1 month but < 3 months), whether they were still doing paid work and how many hours/week (options: 0–8 h, 9–15 h, 16–24 h, 25 h or more). All these data were collected to evaluate their suitability as future outcome measures. Baseline questionnaires were completed at the face-to-face appointments. Follow-up questionnaires were posted to allow participants to complete and return the form at their leisure in a reply-paid envelope. If no response was received after four weeks, a reminder letter and a further copy of the questionnaire were sent. On receipt of each completed questionnaire, a shopping voucher (amount £10) was sent to the respondent.

### Analysis

Descriptive statistics were used to summarise the proportion of people identified in primary care who were eligible for the study and the proportion that agreed to take part. Baseline characteristics were also summarised using counts and percentages for categorical data and means (standard deviations) and medians (interquartile ranges) for continuous data for the whole group of participants and separately for each allocation arm in the trial. Analyses were carried out using Stata version 12.

### Employers’ perspective

To complete this pilot trial, employers who had provided a work placement for at least one participant in the IPS arm were asked to consent to an informal interview with the PI and Study coordinator. Those interested were visited at a time of their choosing in their work environment. Enquiry was made as to their motivation for providing the placement and their reflections on the success (or otherwise) of doing so.

## Results

The pilot study was conducted between November 2017 and September 2019. In total, nine GP practices covering a population in excess of 200 000 people agreed to try to identify eligible participants through primary care using the range of approaches.

### Identification and recruitment

Table 1 summarises the results of recruitment using the different methods. As required by the study protocol, primary care-based methods were evaluated extensively initially. For example, study information packs were posted to 1017 people based on Read code searches but this method yielded only 57 enquiries amongst whom more than half did not meet the eligibility criteria (either retired, already in paid work but struggling to cope because of pain (*n* = 10), or no longer had chronic pain). A further five individuals returned a reply slip to the research team, expressing interest, but were not contactable (incorrect contact number was provided or no response was obtained despite multiple messages left by the study coordinator). A research-active GP who carried out opportunistic screening during appointments identified five eligible participants over 6 months, all of whom were recruited. Likewise, when a dedicated research nurse hand-searched patient notes at one practice for potentially eligible patients and then telephoned those identified, six eligible participants were identified and successfully recruited. However, this proved to be a laborious and resource-intensive approach, taking approximately two weeks of research nurse time. Posters advertising the study were displayed in all nine practices but yielded only two enquiries from patients, both of which came after recruitment closed.

**Table 1.** Methods of recruitment of patients unemployed with chronic pain through primary care and community-based pain services and their success in the pilot trial

Recruitment method	Number approached	Number identified but ineligible	Recruited
Database search (GP) and mailshot	1017	31	26
Hand-searching GP records and telephone contact	6	0	6
Posters in GP surgeries	Unknown	2	0^*^
Opportunistic (GP) during consultations	5	–	5
Opportunistic (pain services) during consultations	13	–	13
Total			50

* Two patients self-referred having seen the posters but after closure of recruitment.

All recruitment methods in primary care proved time-consuming and resource-intensive. Since IPS was only available for a fixed period of time, with the agreement of our Trial Steering Committee, we also opened up recruitment through community-based pain services, using a targeted face-to-face approach. Over a period of five months, this method yielded a further 13 eligible participants who were all recruited and enabled us to complete the pilot trial. Ultimately, a total of 50 individuals were recruited to the pilot study.

### Randomisation

Given the delays to recruitment, block randomisation was compromised by the availability of the IPS intervention (limited to a fixed time point of 30 September 2019, and thus, the final recruit to the active arm was enrolled by 30 September 2018 to allow 12 months of follow-up). Therefore, after random allocation of the first 18 participants, we subsequently allocated as many participants as possible to IPS until the end of September 2018, and thereafter, participants were allocated to TAU. Importantly, all those recruited from one large practice (serving 20 000 people) at the start of the trial (*n* = 13) were randomly allocated 1:1. Ultimately, 28 participants received TAU and 22 received IPS. No one in either arm expressed dissatisfaction with either randomisation or their allocation, nor did anyone withdraw after allocation and before their first appointment.

### Contamination

Although the numbers involved were small, the risk of contamination was assessed to be low. Apart from the single practice in which the GP gave study information packs out during their consultation, GPs were not directly involved with any aspect of the intervention or allocation.

### Characteristics of participants

Table 2 describes the baseline characteristics of those recruited, overall and by allocation. Broadly, those allocated to each arm had similar characteristics: 60% were female, and participants were mostly white. A relatively high proportion were single/divorced (*n* = 21, 42%). The mean age of leaving school was 16 years, and approximately half had gone to further education or university. Less than 10% (*n* = 4) had university degrees. Two participants had never held a paid job. Of the remainder, the majority had previously stopped working mainly or partly for health reasons, including pain but also comorbid mental health/stress and ‘other’ diagnoses. Many participants (*n* = 19, 38%) lived alone, but almost a third had at least one child aged < 18 years and the majority (*n* = 44) had no dependants outside their household. Only 12 owned their home outright, and 68% reported that they were at least ‘just getting by’ financially. Many participants (*n* = 30, 60%) were in receipt of welfare benefits, and 41 (82%) were willing to describe their monthly income. Nearly a third were current cigarette smokers but alcohol abuse was not reported by any. Comorbidities were common, with two-thirds reporting anxiety/depression (and a further three were ‘not sure’). Most participants (*n* = 41) were looking for part-time, rather than full-time work, and wanted < 24 h/week. Nineteen participants had been unemployed < 2 years with a median period of unemployment of 3 years (IQR 1.2–5.5 years), which was similar in both trial arms (2.6 years for IPS and 3.1 years for TAU). Four people (three allocated to TAU and one to IPS) reported a very long period of unemployment prior to baseline (> 20 years).

**Table 2.** Baseline characteristics of all participants and by allocation in the pilot trial

	All (*n* (%))	IPS (*n* (%))	TAU (*n* (%))
Sex			
Males	20 (40%)	10 (45%)	10 (36%)
Females	30 (60%)	12 (54%)	18 (64%)
Ethnic origin			
White	46 (92%)	20 (91%)	26 (93%)
Black-Caribbean	1 (2%)	1 (5%)	0
Black-African	0	0	0
Black-Other	1 (2%)	1 (5%)	0
Indian	2 (4%)	0	2 (7%)
Pakistani	0	0	0
Bangladeshi	0	0	0
Chinese	0	0	0
Marital status			
Married	27 (54%)	8 (36%)	19 (68%)
Single	13 (26%)	10 (45%)	3 (11%)
Civil partnership	0	0	0
Widowed	0	0	0
Divorced	8 (16%)	3 (14%)	5 (18%)
Living with a partner	2 (4%)	1 (5%)	1 (4%)
Age left school (2 missing values)	Mean (SD): 16.0 (1.1) Median (IQR): 16 (15.5–16)	Mean (SD): 15.9 (1.3) Median (IQR): 16 (15–16)	Mean (SD): 16.1 (1.1) Median (IQR): 16 (16–16)
Further education/University			
No	24 (48%)	10 (45%)	14 (50%)
Yes	26 (52%)	12 (55%)	14 (50%)
Educational level			
O Levels/GCSEs (or equivalents)	38 (76%)	15 (68%)	23 (82%)
A Levels (or equivalents)	9 (18%)	3 (14%)	6 (21%)
Vocational training certificate(s)	30 (60%)	10 (45%)	20 (71%)
University degree(s) or HND	4 (8%)	2 (9%)	2 (7%)
Higher professional qualifications	4 (8%)	1 (5%)	3 (11%)
Ever in paid job			
No	2 (4%)	1 (5%)	1 (4%)
Yes	48 (96%)	21 (95%)	27 (96%)
Time since last in paid work			
Median (IQR) (years)	3.0 (1.3–5.5)	2.8 (1.3–4.1)	3.2 (1.3–16.1)
Missing	11	4	7
Leaving job due to health			
No	3 (6%)	1 (5%)	2 (7%)
Yes, mainly due to health	34 (68%)	16 (73%)	18 (64%)
Yes, partly due to health	11 (22%)	4 (18%)	7 (25%)
Missing	2 (4%)	1 (5%)	1 (4%)
Health-related job loss (type of health problem)			
Chronic pain	24 (48%)	14 (64%)	10 (36%)
Back, neck, arm, shoulder or leg	34 (68%)	13 (59%)	21 (75%)
Mental health problem or stress	15 (30%)	5 (23%)	10 (36%)
Heart or lungs	2 (4%)	1 (5%)	1 (4%)
Other	6 (12%)	3 (14%)	3 (11%)
N/A (No HRJL)	3 (6%)	1 (5%)	2 (7%)
Future work prospect			
Part-time	41 (82%)	17 (77%)	24 (86%)
Full-time	9 (18%)	5 (23%)	4 (14%)
Hours in part-time future job			
0–8	10 (20%)	5 (23%)	5 (18%)
9–15	13 (26%)	4 (18%)	9 (32%)
16–24	13 (26%)	6 (27%)	7 (25%)
>25	3 (6%)	2 (9%)	1 (4%)
N/A	9 (18%)	5 (23%)	4(14%)
Missing	2 (4%)	–	2 (7%)
Comorbidities reported			
High blood pressure	No: 36 (72%)		
	Yes: 9 (18%)		
	Not sure: 5 (10%)		
Heart problems	No: 47 (94%)		
	Yes: 3 (6%)		
	Not sure: 0		
Diabetes	No: 46 (92%)		
	Yes: 4 (8%)		
	Not sure: 0		
Kidney disease	No: 49 (98%)		
	Yes: 1 (2%)		
	Not sure: 0		
Previous stroke or ‘TIA’	No: 48 (96%)		
	Yes: 2 (4%)		
	Not sure: 0		
Arthritis	No: 27 (54%)		
	Yes: 19 (38%)		
	Not sure: 3 (6%)		
	Missing: 1 (2%)		
Asthma or other lung problems	No: 37 (74%)		
	Yes: 12 (24%)		
	Not sure: 1 (2%)		
Anxiety or depression	No: 14 (28%)		
	Yes: 33 (66%)		
	Not sure: 3 (6%)		
GI or other stomach problems	No: 37 (74%)		
	Yes: 10 (20%)		
	Not sure: 3 (6%)		

A comparison of participants recruited via primary care with those enrolled through pain services revealed notable differences: those recruited from pain services represented a wider ethnic diversity (31% vs 0% non-white ethnicity); were more likely to be single (54% vs 16%); were more likely to have received further education (69% vs. 46%); had slightly poorer health literacy; had been unemployed longer (median 3.0 years (IQR 3.0–4.1) vs median 1.8 years (IQR: 0.7–6.8); and included the 3 participants who had never worked.

### Retention and questionnaire response rates

60% of participants returned all questionnaires, and these were over-represented in the TAU arm (68% vs 50%). Five (4 in IPS arm, 1 in TAU arm) failed to return any postal questionnaires, and these were mostly men (4/5); white (4/5); single (4/5) and more likely to have undertaken further education (4/5). According to every parameter, they had better health literacy than those who completed at least one postal questionnaire.

### Employment outcomes

The ESWs reported wide variation in how ready participants were for work when first referred. Therefore, amongst the 22 allocated to IPS, eight (36%) attained a job by 12 months of follow-up (including two who moved away to work), a further one was doing voluntary work, four were in vocational training, and seven were actively job-seeking (with one participant having received a job offer), and two were lost to follow-up. In the TAU arm, 8/28 (29%) were in paid work at 12 months, all but one of whom started working within 3 months of recruitment and remained working throughout (the other one was in work by 6 months and remained in work). Amongst the remaining 20, 14 never worked at any point during follow-up and three worked at only one point but stopped again before 12 months of follow-up. Of the remaining three, who only returned one or two questionnaires, they were not working when we did hear from them. A key difficulty in assessing the impact of the intervention on employment was the high rates of non-response to questionnaires.

### Outcome measures for a definitive trial

Overall, when questionnaires were returned, they were well-completed. We evaluated a range of possible outcome measures for completeness and responsiveness to change (Supplementary Table 1), amongst which the measure that appeared most responsive was return-to-work self-efficacy.

### Reflections from employers

Three managers from two SMEs who had hosted a work placement for participants in the pilot trial agreed to be interviewed informally about their experiences. Their reflections are summarised in Table 3. Notably, employers were very positive about employing people with chronic pain and reported seeing their employees gain health improvement.

**Table 3.** Reflections from employers about providing a work placement in the InStep pilot trial

Reflections	Comments /examples as quotations
Glad to have provided the placement	• ‘Giving back to the community’ • Saw it as ‘important to get involved’ • I ‘wanted to give a person unemployed with a health condition a chance’
Benefits to employers of housing a placement	• It had proved easier to obtain an individual prepared to work part-time hours (which was what the Company needed) through this mechanism than on the conventional job market ‘where most people want full-time’. • ‘By involving everyone in the organisation in supporting this employee, it has been good for the business’ and not caused any problems with relationships with other staff.
Cynicism from some senior managers	• ‘The placement was sold to me by senior managers as free labour for 6 months’ but ‘I didn’t see it that way’
Some doubt to begin with	• It was ‘a leap of faith’ when they offered the placement, but ‘it had been well worth the gamble’ in their opinion and said they ‘would not hesitate to offer future work placements if funding became available again. It was brilliant’.
Personal satisfaction	• The two individuals placed here experienced job satisfaction and it was enjoyable to see them ‘flourish at work’
Placement participant may have different needs than other employees	• The employers acknowledged that the employees on work placements tended to ‘need flexibility (including, for example, more sick days) ’ but perceived this was ‘straightforward to accommodate’. ‘They appreciated the support and flexibility’ (eg, with working hours and tasks), ‘which had allowed them to settle in’. • ‘Good communication regularly between the employee and their manager is essential’ • I realised that the pain levels changed and the employee has ‘good and bad days’ but this was easy to accommodate ‘once I understood’ • One worker had been overwhelmed by the job initially, but with support, had been helped ‘to break the job down into manageable tasks’ which had enabled them to cope and eventually to compile a ‘how to’ booklet to help himself and other new recruits to perform the work effectively. • ‘The workers had demonstrated a keen enthusiasm and desire to work’ which had led to them both being taken on permanently after starting the placement.
Benefits to employee in placement	• The employment placement had benefitted the health of their employee: ‘had come off all pain medication within weeks’. • The work gave ‘a sense of worth’ and kept the mind occupied. The employee had told them how much the work helps and that they ‘look forward to coming to work’.
Interview essential	• It was important that they were not obliged to take anybody through this placement scheme but instead were able to interview applicants interested in working with them. • They felt that this was important for the success of the placement and that they would only offer such placements if they could make this selection. • ‘Not all candidates would be physically suited’ (eg, if there were communication issues or a physical disability which simply could not be accommodated in their workplace). • The interview gave an opportunity to assess an individual’s motivation for the work and their motivation was ‘key to the success of the placement’.

## Discussion

This research examined the feasibility of conducting a future definitive RCT investigating the effectiveness and cost-effectiveness of IPS as an intervention to improve health-related quality of life amongst people unemployed with chronic pain, recruiting through primary care. Despite testing a range of different methods in primary care, recruitment of individuals unemployed with chronic pain proved challenging and resource-intensive. Consequently, and in accord with the findings of another UK study (Froud et al., 2020), we have shown that, at least in the UK, a definitive trial would currently not be feasible with the major barrier being the lack of a whole systems approach to health and employment. If appropriate employment support is to be made available to people with pain, there is an urgent need for accurate and up-to-date employment status to be held in primary care records and to improve ability to link health and work data for the future. Indeed, if the role of IPS for people with chronic pain in the UK is to be accurately investigated, this is a fundamental requirement.

Combining recruitment through pain services allowed us to recruit a sample of 50 participants and investigate other feasibility questions. Firstly, chronic pain patients, once identified, were willing to participate, agreed to be randomised and found their allocation, both to IPS and TAU, acceptable. Despite our efforts, few had only recently become unemployed and median duration was 3 years. Primary care recruitment yielded some participants with shorter duration of unemployment (median 1.8 vs 3 years) but the variation was still wide (IQR 0.7–6.8 years). Consequently, many of those who were recruited to the IPS arm were not ‘work ready’, and in some cases, the ESWs reported that participants seemed quite unrealistic about this. Nevertheless, all made some progress towards work during the 12-month follow-up with over a third in paid employment. In the TAU arm, 29% reported that they were in paid work at 12 months, all but one of whom started working by 3 months and remained working throughout. We found that people with long-term unemployment and chronic pain faced significant challenges in terms of work readiness that take time to overcome. Lack of work readiness may reflect problems with the delivery of their chronic pain care such as delays in diagnosis, poor training of healthcare professionals (Donaldson, 2009, British Pain Society, 2012) and/or lack of access to specialist advice when required. Alternatively, it might be the nature of an individual’s journey through a diagnosis of chronic pain, firstly needing a period of adjustment to the ‘incurable’ nature of their problem and also needing to find appropriate therapy for psychological comorbidities. Taking all this into account, it is challenging to know when best a workplace intervention should be offered. Either way, as was suggested in our qualitative work with people with chronic pain (Holmes et al., 2020), follow-up over 12 months may be too soon to fully appreciate the effectiveness of IPS on return to work in a trial, and assessment of employment status at 24 months or longer may be a more appropriate primary outcome, as used by Hellstrom and co-workers in their study of IPS in people with mood and anxiety disorders (Hellstrom et al., 2017).

We experienced difficulty in obtaining responses to follow-up questionnaires (40% non-receipt) despite incentives and reminders, with rates of attrition similar to those seen in a similar study (Froud et al., 2020). Interestingly, in a study of IPS amongst people with moderately severe mental ill-health, only a 60% response to questionnaires was anticipated (Reme et al., 2019). Response rates were poorer amongst those in the IPS arm, particularly those who obtained employment (perhaps perceiving that they were no longer relevant to the trial). This suggests that other methods of data collection would be important in a definitive trial, perhaps using telephone or video calls or a system of text messages. If work commitments limit retention, consideration of data collection methods outside traditional working hours might be necessary. However, returned questionnaires were well-completed and, of the measures investigated, return-to-work self-efficacy appeared to be a measurable, responsive outcome measure (Black et al., 2019). Integration of IPS with pain services proved feasible and has been manualised (Figure 1). Employers who offered work placements for the trial described benefits to the business and the employee and were extremely positive about their involvement.

People unemployed with chronic pain have a number of compounding problems which include reduced self-esteem and self-confidence; progressive loss of fitness and stamina through inactivity; outdated vocational skills; lack of suitable, sustainable employment opportunities; poor availability of ‘tailored’ job-seeking and occupational advice and potential prejudice from employers against people with poor sickness records (Omori, 1997). Recognising this, an aim of this project was to identify people who were relatively recently (minimum 3 months) unemployed. In the UK, people with prolonged unemployment can be identified through Job Centre Plus as most of them will require welfare benefits. As recently unemployed people do not formally register anywhere, it was hoped that we could identify them through primary care. However, this proved difficult. Although primary care electronic software systems include codes for fit notes, they do not have any code for discussion of work or unemployment during a consultation. Moreover, 10-minute consultations (that are specified in the NHS) are already stretched dealing with medical problems and prescriptions to realistically expect lengthy employment discussions. Systems are needed for collecting this information in other ways, for example through data linkage and integrating into electronic healthcare records. It is unrealistic to expect that any system depending on practice staff, GPs or patients to record employment information accurately and in a timely way will be effective. Additionally, there is no agreed system of coding chronic pain (Mansfield et al., 2017). After intensive efforts in one practice, using codes for combinations of medications used to treat chronic pain, a large number of invitations were sent to patients registered with likely chronic pain but this yielded a low number of eligible participants and came at the cost of some complaints to practice staff from patients who were working and thus incorrectly invited to take part.

Previous work by our group (Szplit, 2013) showed that people unemployed with chronic pain can be recruited through pain services, and indeed, it proved successful in this project. A similar methodology has been adopted in a current RCT in Norway (Rodevand et al., 2017, Linnemorken et al., 2018). However, our results, unsurprisingly, suggest that people recruited from this setting are different: they have longer pain journeys; higher rates of comorbidity; represent a more diverse ethnicity and have less work experience so that although return to work can be achieved, it will be more time-consuming and resource-intensive (as seen in the IPS arm). Whilst these are not reasons against providing employment support to people using pain services, earlier intervention would be desirable. Ideally, people would be identified for support whilst they are still in the workplace or as soon as possible after they have lost their job. Employment support at these points will likely be less costly in financial terms and more successful, but new methods of identifying such individuals are urgently needed.

The current research needs to be considered alongside its limitations. It was a pilot trial carried out as part of a programme of work to establish the feasibility of a definitive RCT of IPS for people unemployed with chronic pain. The commissioners (NIHR HTA) were keen that recruitment through primary care was prioritised, and we tested this as rigorously as possible but found it extremely challenging. Given this, we added recruitment from pain clinics to maintain our assessment of feasibility of other aspects of a future trial. Also affected by slow recruitment was the availability of the IPS intervention. In consequence, after initially following our plan of 1:1 block randomisation, we allocated subsequent participants to IPS until it was no longer available and to TAU thereafter, so that this was, as a result, a quasi-randomised study. However, all participants gave consent to random assignment and we experienced no drop-outs after allocation. We asked participants to complete postal questionnaires to assess the feasibility of this approach for obtaining outcome data and identify the most suitable primary outcome but were disappointed with low rates of completion. The primary data reported here are for context, rather than because they were the main purpose of this research. We found that most IPS research does not include the crucial voice of the employers and therefore included their reflections which had been obtained through informal meetings with the study team, but these data were neither collected nor analysed by a qualitative researcher.

IPS has been shown to be effective in improving rates of employment amongst people with severe mental health conditions. The ‘place then train’ model, together with adherence to strictly defined fidelity principles (Drake and Becker, 1996; Drake, 1998; Burns et al., 2007), including most crucially that the participant must want to work, and integration with healthcare provision, have made it a much more effective model of vocational rehabilitation than others. There is good reason to believe that it could be an effective approach for people with a range of other long-term conditions, including chronic pain, and our study adds insight to a growing body of evidence in this patient group. IPS has also been tested amongst other groups, including people with substance misuse conditions (Lones et al., 2017); autism (McLaren et al., 2017); common mental health conditions (Hellstrom et al., 2017; Overland et al., 2017); post-traumatic stress disorder [(Davis et al., 2018); and ex-offenders (Khalifa et al., 2016, Durcan et al., 2018), and this literature has recently been systematically reviewed (Probyn et al., 2021). Additionally, the UK government has undertaken some large-scale government trials and pilots, including people with diverse health conditions (Institute for Employment Studies, 2021). Together, there is a growing body of evidence that IPS can be effective at improving employment for people with a range of health conditions. Sadly, however, our work highlights a significant barrier to undertaking trials such as this in the UK until progress is made towards improved integration of healthcare and employment databases.

## Supplementary material

For supplementary material accompanying this paper visit http://doi.org/10.1017/S1463423622000342. click here to view supplementary material
