# Supplementary material for: A pilot trial investigating the feasibility of a future randomised controlled trial of Individualised Placement and Support for people unemployed with chronic pain recruiting in primary care
Source: Prim Health Care Res Dev. 2022 Jul 22;23:e39. doi: 10.1017/S1463423622000342 (PMC9309751; doi:10.1017/S1463423622000342)
Supplement: Supplementary file 1 [file S1463423622000342sup001.docx]

**Supplementary table 1 Pooled data at baseline and 6-month follow-up for outcome measures amongst 50 pilot participants, data collected to assess optimal outcome for a definitive randomised controlled trial**

|  | **BASELINE** | | | **Range of scores at baseline** | **6-MONTH FOLLOW-UP** | |
| --- | --- | --- | --- | --- | --- | --- |
|  | **No. with complete answers** | **Mean (SD)** | **Median (IQR)** |  | **Mean (SD)** | **Median (IQR)** |
| **Self-efficacy for return to work** | 45 | 53.8 (14.9) | 53 (46-67) |  | 35.7 (14.6) | 35 (23.5-45) |
| **Rosenberg’s self-esteem** | 50 | 16.7 (6.0) | 16 (12-21) | Normal self-esteem 30 (60%)  Low self-esteem 20 (40%) | 16.4 (5.6) | 16.5 (13-20) |
| **WEMWBS** | 49 | 43.1 (10.5) | 41 (37-52) | 14-32: 6 (12%)  33-40: 15 (30%)  41-59: 26 (52%)  60-70: 2 (4%) | 42.5 (9.8) | 43 (36-51) |
| **PHQ-9** | 49 | 11.4 (6.8) | 10 (7-16) | Minimal: 7 (14%)  Mild: 12 (24%)  Moderate: 15 (30%)  Moderately severe: 7 (14%)  Severe: 8 (16%) | 11.5 (7.4) | 10 (6-15) |
| **EQ-5D-5L** | 49 | 0.5 (0.3) | 0.6 (0.3-0.7) |  | 0.6 (0.3) | 0.6 (0.4-0.8) |
| **Your health today** | 50 | 54.0 (21.9) | 50 (40-70) |  | 52.5 (20.9) | 50 (40-70) |
|  |  |  |  | **Mobility**  No problems 5 (10%)  Slight problems 12 (24%)  Moderate problems 20 (40%)  Severe problems 13 (26%)  Unable 0  **Self-care**  No problems 23 (46%)  Slight problems 11 (22%)  Moderate problems 10 (20%)  Severe problems 6 (12%)  Unable 0  **Usual activities**  No problems 4 (8%)  Slight problems 13 (26%)  Moderate problems 19 (38%)  Severe problems 12 (24%)  Unable 1 (2%)  **Pain discomfort**  No pain 1 (2%)  Slight pain 5 (10%)  Moderate pain 23 (46%)  Severe pain 17 (34%)  Extreme pain 4 (8%)  **Anxiety/depression**  Not anxious 10 (20%)  Slightly anxious 15 (30%)  Moderately anxious 20 (40%)  Severely anxious 2 (4%)  Extremely anxious 3 (6%) |  |  |
